# A one-year observational study of all hospitalized acute poisonings in Oslo: complications, treatment and sequelae

**DOI:** 10.1186/1757-7241-20-49

**Published:** 2012-07-24

**Authors:** Cathrine Lund, Per Drottning, Birgitte Stiksrud, Javad Vahabi, Marianne Lyngra, Ivind Ekeberg, Dag Jacobsen, Knut Erik Hovda

**Affiliations:** 1 Department of Acute Medicine, Oslo University Hospital Ullevaal, Kirkeveien 166, Oslo, (0407), Norway; 2 Department of Acute Medicine, Lovisenberg Hospital, Lovisenberggata 17, Oslo, (0456), Norway; 3 Department of Medicine, Diakonhjemmet Hospital, Diakonveien 12, Oslo, (0319), Norway; 4 Department of Medicine, Oslo University Hospital Aker, Trondheimsveien 235, Oslo, (0316), Norway; 5 Department of Medicine, Akershus University Hospital, Sykehusveien 27, Nordbyhagen, (1424), Norway; 6 The National Center for NBC Medicine, Department of Acute Medicine, Oslo University Hospital Ullevaal, Kirkeveien 166, Oslo, (0407), Norwayd

**Keywords:** Antidotes, Mortality, Toxicology, Treatment

## Abstract

**Objectives:**

Changes in poisoning trends may affect both complications and outcomes in patients with acute poisoning. This study reports the treatments given and the frequency of complications, also related to treatment, mortality and sequelae related to various toxic agents.

**Methods:**

All acute poisonings in adults (≥16 years) admitted to the five hospitals in Oslo were included consecutively during one year (2008 to 2009) in an observational cross-sectional multicenter study. A standardized form was completed by the treating physician, which covered the study aims.

**Results:**

There were 1065 admissions in 912 patients. The median length of hospital stay was one day, and 49% were observed in an intensive care unit (ICU). Active treatment was given to 83%, and consisted of supportive therapy (70%), antidote(s) (38%), activated charcoal (16%) and gastric lavage (9%). The most commonly used antidotes were flumazenil (19%), naloxone (17%) and N-acetylcysteine (11%). The rate of treatment-related complications was 2.4% (21/884). Neither flumazenil, naloxone, nor the combination, was associated with convulsions or other complications. Among those receiving N-acetylcysteine, 5% (6/120) developed allergic reactions, one of which mandated discontinuation of treatment. Nineteen percent presented in a coma. Complications developed in 30%, compared with 18% in a 2003 study, mainly respiratory depression (12%), prolonged QTc interval (6%) and hypotension (5%). Eight patients died (0.8%) and five (0.5%) survived with permanent sequelae, mainly anoxic brain damage.

**Discussion:**

Few patients stayed more than two days. The use of the ICU was liberal, considering that only one out of five presented in a coma. Antidotes were frequently given diagnostically. Although N-acetylcysteine induced allergic reactions, most were mild and treatment discontinuation was only necessary once. The frequency of complications had almost doubled in five years, although the poisoning pattern was largely unchanged. However, few patients developed permanent sequelae.

## Background

Acute poisoning contributes to a substantial number of hospital admissions. In the UK, acute poisoning contributed 15–20% of the workload in medical departments in 1999 [1]. Alterations in the poisoning pattern affect clinical features, complications and outcomes, making regular studies mandatory. Similar clinical studies were performed throughout Oslo during 1980 and 2003 [2-5]. In 2003, the incidence was 2.0 per 1000 inhabitants and the outcomes were good, with both low inhospital mortality and sequelae [3].

In 2003, one-quarter of the patients presented in a coma and one-fifth developed complications. Since 2003, poisoning by opioids has increased in Oslo (Lund C, Teige B, Drottning P, Stiksrud B, Rui TO, Lyngra M, Ekeberg O, Jacobsen D, Hovda KE: A one-year observational study of all hospitalized and fatal acute poisonings in Oslo: Epidemiology, intention and follow-up, submitted). Diagnostic use of naloxone and flumazenil is common in Oslo, and this use doubled from 1980 to 2003 [3,5]. As there is a risk of causing unnecessary complications, liberal diagnostic use of flumazenil has been deemed controversial [6]. The 2003 study reported a sixfold increased risk of convulsions in patients receiving both flumazenil and naloxone, but none if they are given alone [3]. In that study, however, the time of antidote administration was not recorded in relation to the onset of complications. As such, they could not be distinguished from complications caused by the poisoning or cerebral hypoxia itself.

For most acute poisonings, treatment is symptomatic, as there is seldom time to wait for a laboratory diagnosis. The poisoned patients presenting in a coma often require urgent diagnosis and management [7], and knowledge of the most common causes is therefore important for clinicians. Local epidemics of special types of poisoning may also impose diagnostic challenges [8]. Data on current treatment practice and the rate of treatment-related complications may reveal important targets for improvement. Furthermore, the rate of both diagnostic uses of flumazenil and naloxone, as well as related complications may have implications, in favor of discontinuation or augmentation of current practice. Knowledge of the toxic substances related to poor outcomes is also important for prevention strategies.

The present study objectives were to study (1) the treatment given and (2) the frequency of complications, also related to treatment. Further objectives were (3) to study the mortality and sequelae and (4) to compare findings with previous studies in 1980 and 2003.

## Material and methods

### Study design

This study was part of a larger one-year observational cross-sectional multicenter study conducted at the outpatient Emergency Medical Agency (EMA, “Oslo Legevakt”), the Institute of Forensic Medicine and the five hospitals in Oslo treating poisoned patients. This paper presents data on their clinical course and treatment in hospitals. Data on the poisoning patterns in hospitals, deaths outside hospitals and on outpatient treatment of acute poisoning are presented separately. For comparative reasons, this study was designed similarly to the studies conducted in 1980 and 2003 [2,5]. In July 2008, there were 466 423 inhabitants ≥16 years in Oslo, the capital of Norway. As Akershus University Hospital treats patients from two municipalities, patients with residency outside Oslo treated in this hospital were excluded.

### Selection of participants

All adults (≥16 years) exposed to a substance in toxic amounts and leading to hospital admission were included consecutively from April 15^th^ 2008 to April 14^th^ 2009. Chronic poisonings were not included. One fatal case of subacute methotrexate poisoning (medication error) and one fatal case of acute renal failure causing subacute metformin poisoning were excluded. In cases where the poisoning was an additional diagnosis, the poisoning had to warrant admission itself. In cases where ethanol and trauma were codiagnoses, or in doubt, a blood alcohol concentration of 54 mmol/L (250 mg/dL) was used as the cutoff.

Of the 912 individuals studied, 460 (50%) were males and the median age was 36 years (range 16–93), 37 in males and 34 in females. The 23 (2.1%) patients who refused to participate did not differ from the studied population in terms of age, gender, poisoning intention or main agents.

### Data collection

All physicians attending poisoned patients in the five hospitals in Oslo participated in the data collection. All evaluations were done by the treating physician who completed a standardized form that included information that satisfied all of the study objectives. Each hospital had a coordinator (medical doctor) monitoring the study. These coordinators manually cross-checked the forms against the electronic patient lists to ensure that all patients meeting the criteria were included. The electronic data journal was used as a supplement where variables were missing. Variables still missing were coded as unknown and excluded from that particular analysis.

Data were manually entered into an SPSS spreadsheet and systematically checked for errors by randomly cross-checking 10% of the variables with the original forms, showing 99.94% consistency.

### Outcome measures

In patients exposed to more than one substance, the main toxic agent was defined as the substance suspected to be the most toxic in the amount assumed taken. This was the treating physicians’ clinical evaluation, based on the information available, such as statements from the patient, companions, ambulance service, clinical observations and laboratory findings. Substances presumed to be less toxic were registered as coagents. In six cases, the main agent was unknown. The median time from exposure to hospital admission was recorded for all patients, based on the information available, except where ethanol was the main toxic agent. This was due to practical difficulties in defining the exact time when the ethanol was ingested. Consciousness was measured at the time of hospital admittance. Coma was defined as <8 and drowsiness as 8–14 on the Glasgow Coma Scale (GCS). Complications were classified according to standard definitions, as in the similar study from 2003 [3], and registered in relation to the treatment given. Hypothermia was defined as body temperature below 35°C (95°F), hypoglycemia as serum glucose level <3.5 mmol/L (63 mg/dL). Respiratory depression was defined as respiratory acidosis (pH < 7.3, pCO_2_ > 7.0 kPa), hypoxia (pO_2_ < 8.0 kPa) or clinical need for ventilation support. Hypotension was defined as systolic blood pressure below 85 mmHg in two subsequent measurements. Conduction disturbances were classified as arrhythmia, but sinus tachycardia was not. Other complications were defined according to general clinical criteria. Each complication was evaluated by the physician as either related or unrelated to the treatment given. Where antidotes had been administered, the exact time for each complication was noted in relation to the antidotes given. Complications arising within one hour after intravenous N-acetylcysteine (NAC) administration and within 10 min after flumazenil or naloxone administration were considered treatment related. Treatment-related complications included among others bronchospasm, hypotension, arrhythmia, convulsions and rashes if not typical of that specific type of poisoning. More unspecific symptoms, such as nausea, vomiting, dizziness, coughing and headache, were not included in this term. Note that neither NAC nor flumazenil was administered in a prehospital setting. The NAC regimen was identical in all hospitals. NAC was administered intravenously according to the Rumack-Matthew normogram in three doses over a period of 21 h [9].

The intention behind the act of poisoning was evaluated by the treating physician. Poisonings with substances of abuse, including heroin and ethanol for recreational purposes, were classified as accidental overdoses with substances of abuse (AOSAs). Suicidal motivation in the act of poisoning was classified as either a possible or definite suicide attempt. This distinction was based on whether the patient considered the toxic agent lethal and whether other measures had been taken to ensure a lethal outcome. If the motive was ambivalent (e.g., by seeking help shortly after ingestion), the poisoning was classified as a possible suicide attempt. Accidents included both external causes of poisoning and self-inflicted accidents (e.g., taking the wrong medication) where the agent had been taken for neither self-harm nor intoxication purposes.

### Statistics

Pearson’s chi-square or Fisher’s exact test (cell values <5) were used to compare frequencies. Age comparisons were done using Mann–Whitney *U*-test.

Univariate logistic regression was used to analyze the association between complications and antidotes given. Univariate and multivariate logistic regression were used to identify factors associated with intensive care treatment. All variables included in the multivariate model were clinically relevant and had a p value <0.20 in the univariate analysis. The assumptions underlying multivariate logistic regression analyses were checked and found to be adequately fulfilled. Findings with p values <0.05 were considered statistically significant. SPSS 16.0 (SPSS Inc., Chicago, IL) was used to perform the statistical analyses.

### Ethics

This study was approved by the Norwegian Regional Ethics Committee and the National Data Inspectorate. Studying intoxicated patients is difficult, including acquiring written consent, because of the nature of their behavior and physical condition. The study participants were therefore informed about the aims of the study and were given a written information leaflet with the name and phone number of the study coordinator. They were also given the right to refuse participation or to withdraw consent at any time without reprisal. Thereafter, their verbal consent was confirmed by the independent interviewing physician in a specific question included in the standardized interview form.

## Results

**Figure 1:**
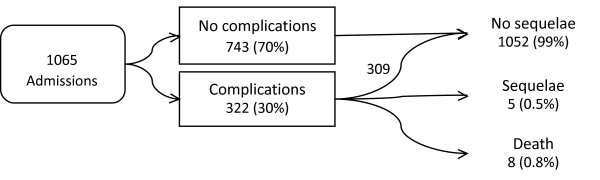
Complications and outcomes in acutely poisoned adults admitted to Oslo hospitals during one year 2008–2009.

During the study year, 1065 admissions were recorded in 912 individuals (Figure 1), contributing to 1954 hospital days (median 1, range <1–48). Seventy-five percent were discharged after less than 24 h, 11% stayed 24–72 h, while 14% stayed more than 72 h. Of the 519 (49%) patients treated in an ICU, only 78 (15%) stayed more than 24 h. Factors associated with ICU treatment were coma (odds ratio 6, CI 4–10), a suicidal intention (OR 2, CI 1–3) and neuroleptics as the main agent (OR 2, CI 1–4) (Table 1). The presence of complications or coagents was a nonsignificant factor. The time from exposure to admission was recorded in 611 (70%) of the cases where ethanol was not the main agent and the median time was 3.0 h (interquartile range 1.5–5.0).

**Table 1:** Factors associated with admission to an intensive care unit among patients with acute poisoning in Oslo during one year 2008–2009: Results of multivariate logistic regression analysis

			**Crude**	**Adjusted**		
	**Total**	**ICU**	**OR**	**95% CI**	**OR**	**95% CI**
**Main agents**						
Other agents^1^	277	119 (43)	Ref			
Ethanol	194	85 (44)	1.0	0.7–1.5	1.0	0.6–1.5
Opioids	114	66 (58)	1.8*	1.2–2.8	1.2	0.7–2.1
GHB	85	50 (59)	1.9*	1.2–3.1	0.9	0.5–1.7
BZD	159	77 (48)	1.2	0.8–1.8	0.9	0.6–1.4
Paracetamol	117	47 (40)	0.9	0.6–1.4	0.8	0.5–1.3
Neuroleptics	60	39 (65)	2.5*	1.4–4.4	2.1*	1.2–3.9
Antidepressants	59	36 (61)	2.1*	1.2–3.7	1.5	0.8–2.8
**Coagent**						
No	593	320 (54)	Ref			
Yes	472	199 (42)	0.6*	0.5–0.8	0.8	0.6–1.0
**GCS**						
Awake (15)	508	194 (38)	Ref			
Drowsy (8–14)	356	166 (47)	1.4*	1.1–1.9	1.3	1.0–1.8
Comatose (<8)	201	159 (79)	6.1*	4.2–9.0	6.0**	3.8–9.5
**Complications**						
No	744	322 (43)	Ref			
Resp. depression	128	87 (68)	2.8*	1.9–4.1	1.4	0.9–2.2
Other complications	193	110 (57)	1.7*	1.3–2.4	1.4	1.0–1.9
**Intention**^**2**^						
Accidents	170	60 (35)	Ref			
AOSA	399	191 (48)	1.7*	1.2–2.4	1.1	0.7–1.7
Suicide attempt	495	267 (54)	2.1**	1.5–3.1	1.8*	1.2–2.7
**Total cases**	1065	519 (49)				

*AOSA* accidental overdose with substances of abuse, *BZD* benzodiazepines, *GCS* Glasgow Coma Scale, *GHB* gamma-hydroxybutyric acid.

*Significant, p < 0.05; **significant, p < 0.001.

^1^Main agents was unknown in six cases.

^2^One patient with unknown intention and one with unknown GCS score were excluded from this analysis.

Few patients (n = 181, 17%) received no further treatment other than observation (Table 2). Supportive treatment was given to 747 patients (70%), antidote(s) to 407 (38%) and activated charcoal to 167 (16%). Gastric lavage was performed in 93 (9%) cases, of which 75 were performed within two hours of ingestion. Intubation was performed in 40 (4%) and mechanical ventilation in 36 (3%) with a median length of three days (range <1–18 days). Noninvasive mask ventilation was performed in 33 (3%), of which 21 were performed during transport by the ambulance service. Twelve patients underwent cardiopulmonary resuscitation, including two patients with respiratory arrest and pronounced bradycardia. All resuscitated patients had return of spontaneous circulation (ROSC). Of the six patients treated with hemodialysis, this was done for elimination purposes in three patients (one case of ethylene glycol and two cases of lithium poisoning) and in the rest for acute renal failure. The sole indicator for forced alkaline diuresis was rhabdomyolysis. Of the 15 patients treated with hyperbaric oxygen (HBO), one was because of gangrene complications; the others had severe CO poisoning.

**Table 2:** Treatment for the most common main toxic agents causing acute poisonings in Oslo during one year 2008–2009

	**Ethanol**		**Opioids**		**GHB**		**BZD**		**Paracetamol**^**1**^		**Other**		**Total**	
**Treatment**	**n**	**%**	**n**	**%**	**n**	**%**	**n**	**%**	**n**	**%**	**n**	**%**	**n**	**%**
Observation only	41	(21)	10	(9)	16	(19)	30	(19)	7	(6)	77	(19)	181	(17)
Treatment*	153	(79)	104	(91)	69	(81)	129	(81)	110	(94)	319	(81)	884	(83)
Total	194^2^	(100)	114	(100)	85	(100)	159	(100)	117	(100)	396	(100)	1065	(100)
Antidote(s)	35	(18)	81	(71)	41	(48)	74	(47)	99	(85)	77	(19)	407	(38)
*-Flumazenil*	28	(14)	24	(21)	33	(39)	65	(41)	5	(4)	52	(13)	207	(19)
*-Naloxone*	18	(9)	79	(69)	38	(45)	26	(16)	7	(6)	18	(5)	186	(17)
*-N-acetylcysteine*	5	(3)	3	(3)			5	(3)	96	(82)	11	(3)	120	(11)
*-Physostigmine*					1^3^	(1)	4^3^	(3)			3^4^	(1)	8	(1)
*-Phytonadione*											4	(1)	4	(<0.5)
*-Hydroxocobalamin*											2	(1)	2	(<0.5)
*-Biperiden*											2	(1)	2	(<0.5)
*-Other*					1	(1)					5	(1)	6	(1)
Other treatment	152	(78)	87	(76)	60	(71)	114	(72)	84	(72)	304	(77)	801	(75)
*-Supportive*	149	(77)	84	(74)	60	(71)	105	(66)	75	(64)	274	(69)	747	(70)
*-Activated charcoal*	3	(2)	6	(5)			24	(15)	36	(31)	98	(25)	167	(16)
*-Gastric lavage*	3	(2)	3	(3)			14	(9)	20	(17)	53	(13)	93	(9)
*-Intubation*	2	(1)	9	(8)	5	(6)	3	(2)	2	(2)	19	(5)	40^5^	(4)
*-Mech. ventilation*	2	(1)	9	(8)	4	(5)	3	(2)	1	(1)	17^6^	(4)	36	(3)
*-HBO*			1^7^	(1)							14	(4)	15	(1)
*-Resuscitation*	1^8^	(1)	6	(6)	1^8^	(1)	2	(1)			2	(1)	12^9^	(1)
*-Therapeutic hypothermia*			6	(6)	1	(1)	2	(1)			2	(1)	11	(1)
*-Forced alk. diuresis*	1	(1)	3	(3)							3	(1)	7	(1)
*-Hemodialysis*	1	(1)	1	(1)							4	(1)	6	(1)

*Alk* alkalic, *BZD* benzodiazepines, *GHB* gamma-hydroxybutyric acid, *HBO* hyperbaric oxygen, *Mech* mechanical, *Paracet* paracetamol.

Supportive treatment consists of iv fluid, warming, nonantidote medication, and supportive care.

Antidotes given on only one occasion are listed as other.

*Including antidote(s) and supportive treatment.

^1^ Including paracetamol–codeine combination drugs.

^2^ 61 (31%) patients had taken coagents.

^3^ Scopolamine coagent, or suspected scopolamine poisoning, but not verified according to criteria.

^4^ Scopolamine poisoning.

^5^ Four patients were intubated to secure airways during gastric decontamination.

^6^ Four cases of antidepressants, three of CO/fire smoke, three of neuroleptics, two of insulin, two of lamotrigine, one amlodipine, one amphetamine, and one case of cocaine poisoning.

^7^ Gangrene.

^8^ Respiratory arrest only and severe bradycardia.

^9^ Of which two had respiratory arrest. All had return of spontaneous circulation.

Flumazenil was given to 207 (19%): 41% of the cases with benzodiazepines as the main agent, 25% of the patients with benzodiazepines as a coagent, and in 33% of the cases with benzodiazepine-like substances as the main agent. Naloxone was given in a prehospital setting, or in the hospital, to 186 (17%): 69% of the cases with opioids as the main agent and 37% of the cases with opioids as a coagent. In 101 (54%) cases, naloxone administration did not improve the level of consciousness, thus weakening the suspicion of opioid poisoning. Flumazenil was given in 86 (42%) cases without an effect on consciousness, weakening the suspicion of benzodiazepines and their derivatives as the main toxic agents. Intravenous NAC was administered in 120 (11%) cases, of whom 96 (80%) had paracetamol as the main agent. A NAC dose regimen was registered in 82 (68%) of the cases; one dose was given in 39%, two in 16% and a full dosage regimen in 45%. NAC was primarily stopped early because of low s-paracetamol levels. The use of physostigmine (n = 8, 1%) was associated with an epidemic of scopolamine poisoning [8]. More than one antidote was given to 116 (11%) patients.

Of the 884 patients receiving treatment, 21 (2.4%) developed complications classified as treatment related (Table 3). Six patients experienced complications after intravenous NAC administration. Five patients (5/120, 4.2%) experienced an urticarial rash and another developed severe bronchospasm (1/120, 0.8%), causing discontinuation of the NAC regimen. Seven patients developed convulsions related to naloxone and/or flumazenil administration. In total, 36 (3.4%) patients had convulsions: 25/782 (3.2%) of those receiving neither flumazenil nor naloxone, 2/96 (2.1%) of those receiving flumazenil alone, 1/74 (1.4%) of those receiving naloxone alone and 4/109 (3.7%) of those receiving both. Four patients experiencing convulsions prior to antidote administration were excluded from this comparison. Pearson’s chi square test did not reveal any significant difference in the rate of convulsions between any of these groups. Two cases with aspiration pneumonia were classified as treatment related after gastric lavage (nonintubated patients). However, the rate was lower than in those not lavaged.

**Table 3:** Treatment-related complications in 21 out of 884 admissions in Oslo during one year 2008–2009

**Treatment**	**Complication**	**n**	**% of treated**
N-acetylcysteine			5%
n = 120	Urticaria	5	
	Bronchospasm	1	
Flumazenil and naloxone			4%
n = 109^1^	Convulsions	4	
Naloxone			3%
n = 74^1^	Convulsions	1	
	Allergic reaction	1	
Flumazenil			2%
n = 96^1^	Convulsions	2	
Gastric decontamination			2%
n = 93	Aspiration pneumonia	2	
Diazepam^2,3^			
	Respiratory depression	2	
	Hypotension	1	
	Aspiration pneumonia	1	
Penicillin^3,4^			
	Allergic reaction	1	
**Total**		**21**	

^1^ Four patients with convulsions prior to flumazenil and/or naloxone administration were excluded.

^2^ Treatment of convulsions.

^3^ Antibiotics and diazepam were not routinely registered.

^4^ Treatment of complications.

On presentation, 508 (48%) were awake, 356 (33%) were drowsy and 201 (19%) were comatose. GHB was the agent that most often caused coma (26%, 52/201), followed by ethanol (20%, 40/201) and opioids (17%, 35/201) (Table 4). Coma was most common in poisoning with GHB (61%, 52/85), cocaine (38%, 5/13) and opioids (31%, 35/114). Only 14% of women presented in a coma, whereas 23% of males were comatose (p < 0.001).

**Table 4:** Toxic agents causing coma and the most common toxic agents in 201 comatose patients with acute poisoning in Oslo during one year 2008–2009

**Main agent**	**% in a coma**	**n (%) of coma**
GHB	(61)	52 (26)
Ethanol	(21)	40 (20)
Opioids	(31)	35 (17)
BZD	(18)	28 (14)
Cocaine	(38)	5 (2)
Antidepressants	(8)	5 (2)
BZD-like substances	(9)	5 (2)
Neuroleptics	(8)	5 (2)
Paracetamol	(4)	5 (2)
Others	(10)	21 (10)
Total		201 (100)

*BZD* benzodiazepines, *GHB* gamma-hydroxybutyric acid.

Complications developed in 322 patients (30%): one in 208 (20%), two in 74 (7%) and three or more in 40 patients (4%) (Table 5). In 282 (26%) cases, the complications were already present on admission. The most frequent complications were respiratory depression (n = 129, 12%), prolonged QTc interval (n = 64, 6%), hypotension (n = 50, 5%) and pneumonia (n = 43, 4%). Ten (1%) patients had a cardiac arrest with ROSC, of which four died and three developed permanent brain damage. In total, eight (0.8%) patients developed signs of cerebral damage. The frequency of complications was highest for opioids (56%), tricyclic antidepressants (54%) and cardiovascular drugs (50%), while the mean number of complications per admission was highest for tricyclic antidepressants (mean 1.3).

**Table 5:** Complications and toxic agents in 1065 cases of acute poisoning in Oslo during one year 2008–2009

	**Opioids**	**TCA**	**Cardiovascular drugs**	**Amphet-amine**	**Cocaine**	**GHB**	**Neuro-leptics**	**Other anti-depressants**	**BZD**	**Ethanol**	**BZD-like substances**	**CO/fire smoke**	**Paracet-amol**^**1**^	**Others**	**Total**	**% of total**
n poisonings	114	13	18	25	13	85	60	46	160	194	58	48	116	115	1065	
n with complications	64	7	9	12	6	35	17	13	41	48	14	8	16	32	322	
% with complications	56^2^	54	50	48	46	41	28	28	26	25	24	17	14	28	30	
Mean n of complications per admission	1.1	1.3	0.9	0.8	0.8	0.6	0.5	0.4	0.4	0.3	0.4	0.2	0.2	0.4	0.5	
% observed in ICU	58	85	17	36	54	59	66	54	48	44	56	35	41	43	49	
Respiratory depression	41	3	2	4	4	16	3	2	20	13	9	5	2	5	129	12
Prolonged QTc	9	3		1		7	8	5	8	10	2		2	9	64	6
Hypotension	9	1	2	2		4	3		5	12	4		3	5	50	5
Pneumonia	12	1	1	1	1	2	3	2	4	5	4	2	1	4	43	4
Convulsions	7	2	1	3	1	3	5	5	1	4			1	3	36	3
Arrhythmia	6	2	6	1		9			1	2		1	3	4	35	3
Hypothermia	6	1		2		6	1		5	10			2	1	34	3
Rhabdomyolysis	8				1		1	1	3	2			1	1	18	2
Acute renal failure	6	1	2	2	1				1	1			2	1	17	2
Cardiac arrest ROSC	6				1				2					1^3^	10^4^	1
Allergic reaction	1					1			1	2			6^5^		11	1
Prolonged QRS	1	3					1		2					2	9	1
Cerebral damage	3				1	1						1	1	1^6^	8^7^	1
Hypoglycemia	2								3					2	7	1
Others	7		2	3		1	4	2	4		3	2	4	5	37	3

*BZD* benzodiazepines, *GHB* gamma-hydroxybutyric acid, *ICU* intensive care unit, *ROSC* return of spontaneous circulation, *TCA* tricyclic antidepressants.

^1^ Including a paracetamol–codeine combination drug.

^2^ 77% among methadone.

^3^ After inhalation of propane and Freon gas in a car.

^4^ Of which four died and three developed permanent cerebral sequelae.

^5^ Allergic reaction to intravenous N-acetylcysteine administration.

^6^ Suicide attempt with insulin.

^7^ Of which two patients died and three developed permanent sequelae.

Of the eight patients (0.8%) who died, three died within 24 h of admission (Table 6). In three of these patients, intensive care treatment was discontinued because of futility/severe anoxic brain damage. The mortality among those presenting in a coma and those requiring mechanical ventilation was 4.0% (8/201, p < 0.001) and 22.2% (8/36, p < 0.001), respectively. Five (0.5%) patients survived with permanent sequelae. All were male and the median age was 39 (range 23–48). Four suffered anoxic brain damage following cardiac arrest, while one patient developed mitral valve endocarditis requiring surgery after an overdose with intravenous (contaminated) heroin (Table 7).

**Table 6:** Fatal acute poisonings in Oslo during one year 2008–2009

**Sex, age**	**Main agent**	**Coagents**	**Intention**	**Days**	**Days on ventilator**	**Comments**
♂ 25*	Heroin	Ethanol	AOSA	6	6	CA (A) with ROSC. Anoxic brain damage.
♀ 25	Heroin		AOSA	4	4	CA (A) with ROSC. Anoxic brain damage.
♀ 29	Insulin	Ethanol	Definite suicide	9	9	Hypoglycemic brain damage. Organ donor.
♀ 38	Methadone	Heroin, BZD	Possible suicide	<1	<1	CA (A) with ROSC, long time anoxia.
♂ 45	Heroin	Ethanol	AOSA	<1	<1	CA (A) with ROSC, circulatory failure.
♀ 75*	BZD		Accident, dementia	33	17	Coma, hypotension, respiratory failure.
♀ 77	Amlodipine	BZD-like substances	Definite suicide	1	1	Coma, acute renal failure, IABP, circulatory failure.
♀ 86*	CO		Definite suicide	<1	<1	Coma, third-degree burn (25% of skin), HbCO 26%, respiratory failure.

*Active treatment discontinued.

*AOSA* accidental overdose with substances of abuse, *A* asystolia, *BZD* benzodiazepines, *CA* cardiac arrest, *HbCO* carboxyhemoglobin fraction, *IABP* intra-aortic balloon pump, *ROSC* return of spontaneous circulation.

**Table 7:** Acute poisonings resulting in permanent sequelae in Oslo during one year 2008–2009

**Sex, age**	**Main agent**	**Coagents**	**Intention**	**Days**	**Days on ventilator**	**Comments**
♂ 23	GHB		AOSA	9		CA (A). Pneumonia, generalized status myoclonicus, cerebral edema. Severe cerebral damage.
♂ 25	Methadone		Definite suicide attempt	18	7	Drug abuse. CA (A). Mild anoxic cerebral damage.
♂ 27	Heroin		Definite suicide attempt	14	13	Drug abuse. Respiratory depression. Mild anoxic cerebral damage.
♂ 44	Cocaine	BZD, anabolic steroids, paracetamol	AOSA	7	3	CA (VF). Pneumonia, rhabdomyolysis and acute renal failure. Mild cerebral damage.
♂ 48	Heroin		AOSA	17		Drug abuse. Pneumonia, acute renal failure and mitral valve endocarditis requiring surgery. Prosthetic mitral valve.

*A* asystolia, *AOSA* accidental overdose with substances of abuse, *BZD* benzodiazepines, *CA* cardiac arrest, *GHB* gamma-hydroxybutyric acid, *VF* ventricular fibrillation.

## Discussion

Although 75% of the patients were discharged after only one day, as many as 49% were observed in an ICU. Nineteen percent presented in a coma, most frequently caused by GHB or ethanol. Thirty percent developed complications, compared with only 18% in 2003, and the rate was highest for opioids and tricyclic antidepressants. Seventy-five percent received treatment other than observation. Intravenous NAC was the only antidote associated with treatment-related complications, and most were mild. Five (0.5%) developed permanent sequelae, of which three had anoxic brain damage. The inhospital mortality rate was 0.8% and corresponds to similar studies [3,5].

### Treatment

The proportion of patients receiving active treatment and antidotes was similar to the 2003 study [3]. The proportion receiving naloxone increased slightly from 2003 (14% vs. 17% in 2008, p = 0.03) reflecting an increase in opioid poisonings (7% vs. 11% in 2008) (Lund C, Teige B, Drottning P, Stiksrud B, Rui TO, Lyngra M, Ekeberg O, Jacobsen D, Hovda KE: A one-year observational study of all hospitalized and fatal acute poisonings in Oslo: Epidemiology, intention and follow-up, submitted). The proportion receiving flumazenil declined slightly (23% vs. 19% in 2008, p = 0.04), although the fraction of poisonings by benzodiazepines was unchanged. Interestingly, the proportion of patients receiving flumazenil diagnostically increased from 23% to 42%, although the total use declined. Thus, patients who were not benzodiazepine intoxicated were more likely to receive flumazenil than before, whereas those who had taken benzodiazepines were less likely to be treated with the antidote, probably because those patients were circulatory and respiratory stable and had a better defined diagnosis. The liberal diagnostic use of flumazenil was addressed after the 2003 study, but obviously with little effect.

Although still high, the rate of gastric lavage was almost halved compared with the 2003 study (17% vs. 9% in 2008) [3]. In comparison, studies from the Netherlands and the UK showed lavage rates of 2% and zero [10,11], reflecting local differences in treatment policies, and indicating overuse of this procedure in Oslo. According to international guidelines, there is no evidence for gastric decontamination more than 1–2 h after intake, except for intake of very toxic agents, or agents with anticholinergic effects delaying gastric emptying [12,13]. As such, it is surprising that one-fifth of these procedures were performed after this time frame. In our opinion, there is clinical evidence for gastric decontamination in the most severely poisoned patients (based on a clinical evaluation of complications and overall clinical condition), also with a larger time window between apparent time of ingestion and hospitalization, and such practice is often associated with tablet content removed from the stomach. A probable explanation for this may be delayed gastric emptying, well known to be associated with heavy ethanol intake [14]. We have only found indirect support for this in the literature, as the most severely poisoned patients are often excluded from studies on gastric decontamination. As these patients accounted for a small proportion in this study, the high use of gastric decontamination should be assessed.

It may seem somewhat surprising that the majority of ethanol poisonings received active treatment. However, this consisted almost solely of supportive intravenous fluids. Only 31% of these patients were exposed to multiple agents.

### Treatment-related complications

The rate of treatment-related complications was low (2.4%) and the only significant correlation was for intravenous NAC, which induced allergic reactions in 5% of cases. Only one case of bronchospasm mandated treatment termination, most probably related to the initial bolus dose given over one hour. Intravenous NAC has previously been correlated to allergic reactions at rates of 3–6% [15,16], similar to the present study (5%).

Neither naloxone nor flumazenil, alone or in combination, could be linked to an increased rate of complications, such as convulsions, although this has been shown in previous studies [6,17]. Although the diagnostic use was liberal, it is likely that physicians did not administer any antidotes under circumstances where they were considered contraindicated. The 2003 study showed an increased rate of convulsions among patients receiving flumazenil and naloxone in combination, but there was no information about the onset of complications in relation to the time of antidote administration [3]. This makes it difficult to determine whether the complications were caused by the antidotes given or if they were merely correlated to the poisoning itself. Poisoning may cause convulsions through respiratory depression and hypoxia [18]. As an illustration, the four patients that developed convulsions prior to antidote administration were hypoxic. Consequently, hypoxia may seem to be the main cause of convulsions in this study. A randomized controlled trial of flumazenil versus a placebo among comatose overdose patients showed no difference in major complications, such as convulsions [7]. Although seemingly safe, the cost-effectiveness of diagnostic use has been questioned [19]. With no significant side effects associated with its diagnostic (over)use, a more restrictive use by doctors wanting a rapid diagnosis in drowsy patients may be difficult to obtain. It has also been argued that this liberal use of flumazenil may reduce the number of cerebral CT scans.

### Complications

Less than half the patients were awake on admission, illustrating the challenge in determining the main toxic agent and hence the usefulness of knowing the current pattern of poisoning in the area. GHB and ethanol were the most common causes of coma on admission, as they were in the 2003 study [3]. Opioids have now replaced benzodiazepines as the third most common cause. The comatose cocaine-poisoned subjects had also taken sedatives. The total proportion of patients presenting in a coma was slightly lower compared with 2003 (23% vs. 19% in 2008, p = 0.02) [3].

Significantly more patients developed complications as compared with 2003 (18% vs. 30% in 2008, p < 0.001) [3]. This increase was mainly seen in terms of “heavy” complications such as respiratory depression (largely due to opioids), pneumonia, hypotension, hypothermia, convulsions and renal failure. This indicates that the present patient population was more severely poisoned than that studied in 2003. Both the observed increase in suicidal motivation and the increase in opioid poisoning may support this (Lund C, Teige B, Drottning P, Stiksrud B, Rui TO, Lyngra M, Ekeberg O, Jacobsen D, Hovda KE: A one-year observational study of all hospitalized and fatal acute poisonings in Oslo: Epidemiology, intention and follow-up, submitted). A possible triage confounder could be the large number (n = 2348) of low acuity patients treated at the outpatient clinic in Oslo (the EMA), which more than doubled from 2003 to 2008 [20,21]. As such, the present hospitalized patients may lack some of the “lighter” poisoned patients, as reflected by the fact that those given observation only—and no active treatment—decreased from 25% to 17% [3]. Prehospital treatment of opioid overdoses is common in Oslo [21]; hence, assuming that the population presenting in hospital is more severely poisoned or is exposed to multiple agents seems logical.

Interestingly, half the patients were observed in an ICU, although only 19% were comatose on admission. In comparison, only 5% were observed in an ICU in a Dutch study and the inhospital mortality was zero [10]. One reason for the extensive use is the triage pattern in Oslo, with the majority of low acuity poisonings treated at the EMA. However, the overall proportion treated in an ICU (15%, 519/3413) was still three times as high as reported in the Dutch study. This indicates overuse of expensive resources. Presence of complications was not associated with ICU treatment, in support of this. Nevertheless, the three predictors for ICU treatment, coma, suicidal intention and intake of neuroleptics, seem relevant from a patient safety perspective. Most of these patients were only observed for a couple of hours in an ICU until they were clarified/stabilized and could be referred to an observation unit. Even so, these patients could probably be treated more cost-efficiently without impacting patient safety.

Antidepressants, both selective serotonin reuptake inhibitors and tricyclic (TCA), are known to cause QTc prolongation in overdose [22]. As such, the observed increase in prolonged QTc is interesting as the number of poisonings with antidepressants was unchanged from 2003. One explanation could be the focus on prolonged QTc as a marker for patients at special risk of developing “torsade de pointes” ventricular tachyarrhythmias. The mean number of complications per admission was highest for tricyclic antidepressants (mean 1.3), as in 2003 [3]. Although newer and “safer” antidepressants are available, the proportion of both prescriptions and acute poisonings with TCA has not declined since 2003 [2,23,24]. This may reflect that severe depressions are still treated with TCA and these patients are the most likely to attempt suicide.

### Mortality and sequelae

Although one-third of the patients experienced complications, the mortality was relatively low and few developed sequelae. The inhospital mortality in Oslo was 0.8%, compared with 1.1% in 2003 and 0.5% in 1980 [3,5]. Both the mortality rate and rate of sequelae are therefore at about the same level as in similar previous studies from Oslo and from other countries [25,26]. However, the increasing number of poisonings treated at the EMA in Oslo with zero mortality indicates that the mortality actually is lower. Note that all patients dying from cardiac arrest had asystolia (Table 6), known to be associated with a poorer prognosis than ventricular fibrillation [27]. Also note that the only young patient who died, who was not a drug abuser, became an organ donor. The main cause of sequelae was substance abuse poisonings in males, mainly by opioids. Methadone poisoning caused one drug-related death, and permanent sequelae in a suicide attempt. This may reflect increasing rates of methadone-related deaths in the Nordic countries in recent years [28,29]. Although increasing fatalities, the number of participants in methadone maintenance treatments in Oslo is increasing (n = 1171 in 2009), hence resulting in a relatively low mortality rate [30]. Of all deaths from opioids in Oslo in 2008, 13% (9/72) were due to methadone (Lund C, Teige B, Drottning P, Stiksrud B, Rui TO, Lyngra M, Ekeberg O, Jacobsen D, Hovda KE: A one-year observational study of all hospitalized and fatal acute poisonings in Oslo: Epidemiology, intention and follow-up, submitted).

### Strengths and limitations

A major strength of this study is that it was conducted prospectively within a defined geographical area. It included unselected data of all acute poisonings throughout a whole year, eliminating bias from seasonal variations. Since similar studies were performed in the same area in 1980 and 2003, comparisons are more reliable than comparing retrospective studies from different—and often undefined—locations and populations.

In terms of limitations, many physicians assisted in the inclusion of patients. This was an advantage when ensuring complete inclusion, but it may have resulted in high interrater variability. A few patients may also have been missed, despite consecutive inclusion, cross-checking of forms against hospital patient lists, and thorough follow-ups.

It is debatable whether a routine verification of the main toxic agents should have been performed to link the complications to the right toxic agents. Moreover, the presence of other agents may have affected these correlations. However, as treatment is mainly symptomatic and not based on laboratory detection of toxic agents, this study was mainly based on the clinical findings and blood/urine tests used in a routine clinical setting. Furthermore, the limited value of laboratory testing in the clinical setting has been demonstrated [31]. The evaluation of treatment-related complications was limited by a small sample size. All available information was used to estimate the time from exposure to hospital admission, as this information would otherwise be difficult to evaluate.

## Conclusions

The frequency of complications had almost doubled in five years, although the poisoning pattern was largely unchanged. The rate of complications was highest for tricyclic antidepressants, opioids and cardiovascular drugs. However, the inhospital mortality was low and few developed permanent sequelae. The most common cause of coma was GHB, ethanol and opioids. The use of an ICU was liberal considering only one out of five patients presented in a coma. Flumazenil and naloxone were frequently given diagnostically and were not correlated to any complications. Although NAC induced allergic reactions, most were mild and did not require treatment discontinuation.

## Abbreviations

AOSA, Accidental overdoses with substances of abuse; EMA, The Emergency Medical Agency in Oslo (“Oslo legevakt”); EMCDDA, European Monitoring Centre for Drugs and Drug Addiction; GCS, Glasgow coma scale; GHB, Gamma-hydroxybutyric acid; ICU, Intensive care unit; NAC, N-acetylcysteine; OR, Odds ratio; TCA, Tricyclic antidepressants; ROSC, Return of spontaneous circulation.

## Competing interests

The authors declare that they have no conflict of interest. The authors alone are responsible for the content and writing of this paper.

## Authors’ contributions

KEH, DJ and OE conceived the study and designed the trial. CL, BT, PD, BS, JV and ML supervised the conduct of the trial and data collection. CL managed and analyzed the data, including quality control, and drafted the manuscript. All authors read and approved the final manuscript.
